# LK6/Mnk2a is a new kinase of alpha synuclein phosphorylation mediating neurodegeneration

**DOI:** 10.1038/srep12564

**Published:** 2015-07-29

**Authors:** Shiqing Zhang, Jiang Xie, Ying Xia, Shu Yu, Zhili Gu, Ruili Feng, Guanghong Luo, Dong Wang, Kai Wang, Meng Jiang, Xiao Cheng, Hai Huang, Wu Zhang, Tieqiao Wen

**Affiliations:** 1 Laboratory of molecular neural biology, School of life sciences, Shanghai University, 333 Nanchen Road, 200444, Shanghai; 2 Institute of systems biology, Shanghai University, 99 Shang Da Road, 200444, Shanghai; 3 School of computer engineering and science, Shanghai University, Nanchen Road, 200444, Shanghai

## Abstract

Parkinson’s disease (PD) is a movement disorder due to the loss of dopaminergic (DA) neurons in the substantia nigra. Alpha-synuclein phosphorylation and α-synuclein inclusion (Lewy body) become a main contributor, but little is known about their formation mechanism. Here we used protein expression profiling of PD to construct a model of their signalling network from drsophila to human and nominate major nodes that regulate PD development. We found in this network that LK6, a serine/threonine protein kinase, plays a key role in promoting α-synuclein Ser129 phosphorylation by identification of LK6 knockout and overexpression. *In vivo* test was further confirmed that LK6 indeed enhances α-synuclein phosphorylation, accelerates the death of dopaminergic neurons, reduces the climbing ability and shortens the the life span of drosophila. Further, MAP kinase-interacting kinase 2a (Mnk2a), a human homolog of LK6, also been shown to make α-synuclein phosphorylation and leads to α-synuclein inclusion formation. On the mechanism, the phosphorylation mediated by LK6 and Mnk2a is controlled through ERK signal pathway by phorbolmyristate acetate (PMA) avtivation and PD98059 inhibition. Our findings establish pivotal role of Lk6 and Mnk2a in unprecedented signalling networks, may lead to new therapies preventing α-synuclein inclusion formation and neurodegeneration.

Parkinson’s disease (PD) is a common age-associated neurodegenerative disease characterized by the loss of dopaminergic (DA) neurons in the substantia nigra and the accumulation of protein aceousintraneuronal inclusions known as Lewy bodies Karuppagounder, Brahmachari^1^,^2^. The clinical and pathological syndromes associated with the familial PD cases are resting tremor, muscular rigidity, bradykinesia, hypo-/akinesia, small handwriting, flexed posture, and postural instability^3^. Previous research in the genes of α-synuclein^4^,^5^ Parkin^6^,^7^ and DJ1^8^,^9^ showed association with autosomal inherited forms of PD, implying these genetic factors lead to PD. However, the exact cause of PD is still unclear. It has been suggested that ubiquitin-proteasome system defect^10^, mitochondrial dysfunction^11^, and oxidative stress^12^ are associated with PD.

Alpha-synuclein, a 140-amino acid protein, is expressed throughout the central nervous system (CNS) and is enriched in presynaptic terminals, lipid membranes, and vesicles^13^. Alpha-synuclein, the major component of Lewy bodies (LB), is a presynaptic protein with possible physiological functions in regulating synaptic plasticity, dopamine neurotransmission, endoplasmic reticulum/Golgi trafficking, and acting as a molecular chaperone^14^,^15^. Two point mutations in the α-synuclein gene (A53T, A30P) which increase the potential for misfolding, oligomerization, and aggregation^16^,^17^ have been associated with some autosomal-dominant PD^4^,^18^, It has been found that α-synuclein oligomers play a key role in disease. Both A53T and A30P oligomerise more rapidly than human wild type α-synuclein^19^. Alpha-synuclein phosphorylation at serine 129 plays a crucial role in promoting the formation of soluble oligomers, aggregates and LB-like cytoplasmic inclusions^20^,^21^,^22^ in the pathogenesis of PD. Studies have indicated that α-Syn is phosphorylated mainly by casein kinases (CK1 and CK2)^23^,^24^,^25^, GRKs (GRK2, GRK3, GRK5, and GRK6)^26^, Polo-like kinase 2^27^ and LRRK2^28^.

In this study, we aim to use a systems biology approach to explore PD pathogenesis induced by α-synuclein mutation in a systematic and integrated view. Based on our experimental data and public database DIP, which contains all information of protein-protein interaction, we constructed a drosophila protein-protein interaction networks (PIN) and further mapped it into a human background by algorithm to construct a human PIN, which provided insight into molecular pathways involved in human PD. Through network analysis, a signal pathway regulated by LK6 phosphorylation is demonstrated. AsLK6 is homologue of human Mnk2a, we further identified that Mnk2a can phosphorylate α-synuclein by the ERK signal pathway, and leads to α-synuclein inclusion (Lewy body) agglomeration in human and mouse neurons, showing that Mnk2a may play a pivotal role in the formation of Parkinson’s disease.

## Results

### Protein Expression Profiles Altered in α-synuclein A30P and A53T Brains

**Figure 1:**
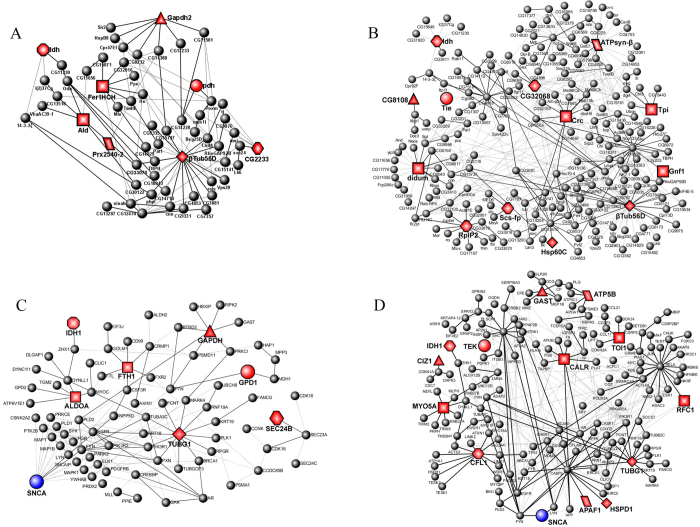
α-synuclein A53T/A30P PIN. Drosophila expressing α-synuclein A53T/A30P related PIN was constructed with our experimental data and the DIP database. (**A**) The network of Drosophila expressing α-synuclein A53T shows 60 proteins and 100 interactions in 20720 interactions from DIP database of Drosophila. The 8 biggest nodes identified by mass spectrometry with red color stand for the corresponding altered proteins in human. (**B**) The network of Drosophila expressing α-synuclein A30P shows 217 proteins and 330 interactions in 20720 interactions from DIP database of Drosophila. The 13 biggest nodes with red color stand for the corresponding altered proteins in human. Nodes highlighted in redare from our experimental data. Broad lines represent the proteins interaction both exist in Drosophila and Human networks. Red nodes with the same shapes represent the same functions both in networks of Drosophila and Human (**C**), The human network in relationship to Drosophila expressing α-synuclein A53T. It shows 83 proteins of 6340 proteins and 146 interactions in 23591 interactions from HPRD database of human. (**D**) The human network in relationship to Drosophila expressing α-synuclein A30P. It shows 161 proteins of 6340 proteins and 316 interactions in 23591 interactions from HPRD data base of human. The 13 biggest nodes with red color stand for the corresponding altered proteins in the Drosophila expressing α-synuclein A30P. The big green node (SNCA) stands for α-synuclein. Broad lines represent that the protein interactions exist in both Drosophila and Human networks. Each node and its counterpart in Drosophila and Human networks are shown with the same color and same shape in these two figures.

**Figure 2:**
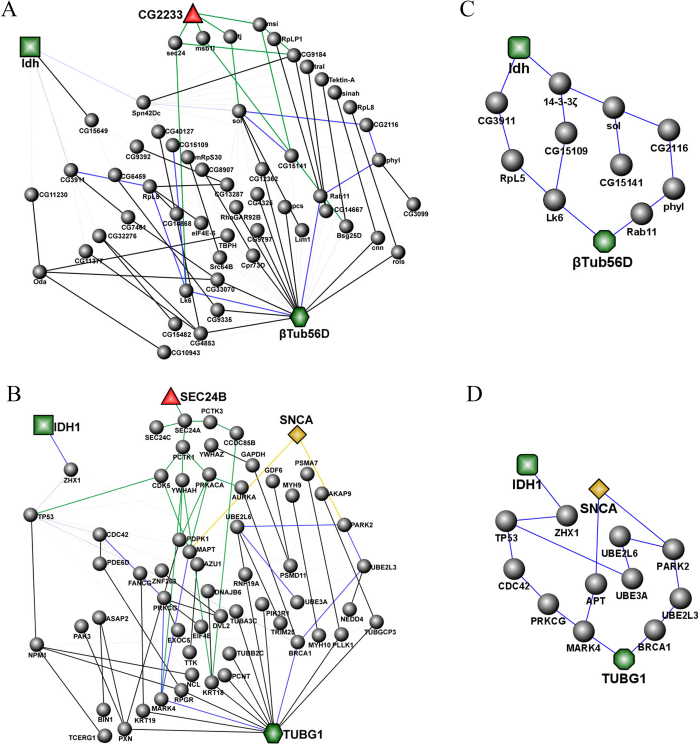
The common interaction networks between β-tub56D and Idh. (**A**) The network between β-tub56D and Idh included 56 proteins and 104 interactions in 20720 interactions from DIP database of Drosophila. (**B**) The network between β-tub56D and Idh included 61 proteins of 6340 proteins and 84 interactions of 23591 interactions from HPRD database of human. Blue lines represent the proteins interaction also exists in Drosophila network and human network. (**C**) Representative graph of two main pathways in Drosophila network. (**D**) Representative graph of two main pathways in Human network. The two main pathways in (C,D) are extremely similar.

To gain a global mapping of the target proteins induced by A30P/A53T, a 2-dimensional electrophoresis (2DE) approach was used to analyze protein expression differences at the proteomic level (S-Fig. 1). After analysis of 3 sets of brains (α-synuclein A30P/A53T and w1118 at day 19 after eclosion) by ImageMaster 2D Platinum 6.0 software, in-gel digestion and mass spectrometry were performed. We limited mass spectrometry analysis to proteins that significantly (p < 0.05) changed more than 2-fold in expression. 35 proteins of about 800 detectable proteins of the Drosophila expressing α-synuclein A30P/A53T met the criterion above and were subjected to mass spectrometry. All identified proteins and their properties are classified and listed in S-table 1 and S-table 2. Surprisingly, In A30P or A53T mutants, we found that the presence of β-tub56D and Idh, (S-table 1 and S-table 2) which was further validated in two genotypes of Drosophilae (S-Fig. 2A–L). β-tub56D is one of the cytoskeletal proteins. A previous study indicated that the aggresomes in PD are surrounded by a tubulin rather than a vimentin cage-like structure, suggesting a close relationship between tubulin and PD^29^. Idh is a mitochondrial protein mainly involved in metabolic processes. Recent reports indicated that energy metabolism enzymes, including isocitrate dehydrogenase, are implicated in protecting cells from α-synuclein in neurodegeneration^30^,^31^. Meanwhile, CG2233, an uncharacterized protein in biological function, is found in a decreased abundance in transgentic α-synuclein A53T Drosophila brains (S-Fig. 2M–O), suggesting a potential connection with α-synuclein function.

### The protein-protein interactions network reveals the protein related α-synuclein mutation

To explore the relationship between the proteins above identified in α-synuclein A30P and A53T mutants, we constructed PIN based on our experiments and the DIP database. Fig. 1A shows the interaction network composed of 60 proteins from 7038 proteins in the database, in which eight proteins found in our experiment (highlighted in red) appear in the inter-linkages in the A53T. Fig. 1B also indicates the interaction network composed of 217 proteins from 7038 proteins in the database, in which 13 kinds of protein interactions were induced by the A30P mutation. Both A53T and A30P PIN revealed the roles and relations of these proteins in a systematic and integrated view. It should be pointed out that there is no α-synuclein in drosophila in a natural state, and thus α-synuclein does not exist in the drosophila database. So there is no α-synuclein in Fig. 1A,B.

Given that Drosophila is only a model organism, we would like to use it to answer questions in human. We therefore made a human PIN prediction based on the drosophila PIN to understand the relationship between fly and human. By mapping the drosophila networks into the human background, we constructed the human networks shown in (Fig. 1C,D), corresponding to the α-synuclein A30P and A53T mutants. The data summarized in S-table 3 and S-table 4 indicate the corresponding relationships of the proteins in networks of α-synuclein A53T (Fig. 1A,C) and α-synuclein A30P (Fig. 1B,D), suggesting that α-synuclein PINs possibly serve as a useful reference for a better understanding of PD pathology.

### Co-expression network deriving from α-synuclein mutations

To extract the co-features deriving from different α-synuclein mutations, we firstly looked for the co-expression proteins in α-synuclein A53T and A30P. From analysis of protein expression, we noticed that the a fore mentioned proteins β-tub56D and Idh were jointly involved in PINs both in Fig. 1A,B. We therefore focused on investigating the linkages between β-tub56D and Idh. The resulting network is shown in Fig. 2A, which is a common network for A53T and A30P mutants. More information was showed in S-Table 5. In support of this information, a human PIN was constructed as shown in Fig. 2B in which α-synuclein (SNCA, highlighted in yellow) directly links PARK2 and MAPT. This is fully consistent with previous reports, i.e. SNCA, PARK2 and MAPT are closely associated with Parkinson’s disease^32^,^33^. We are here for the first time to clarify their relationships at the network level.

More valuably, by comparing Drosophila and human networks (Fig. 2A,B), we could find their similarities (shown in node color and shape) through counterparts between Drosophila and human. β-tub56D in drosophila (Fig. 2A) is represented by a green diamond, it’s human counterpart TUBG1 (Fig. 2B) is the same color and shape; other nodes like Idh and IDH1, CG2233 and SEC24B are also the same corresponding to drosophila and human. We further found the similarity of the networks between Drosophila and human highlighted in blue in Fig. 2A,B. Extracting links highlighted in blue from Fig. 2A,B, we respectively obtain a clearer and contracted drosophila (Fig. 2C) and human network (Fig. 2D) involved in the MAPK pathway (β-tub56D-LK6-Rpl5-CG3911-Idh and β-tub56D-LK6-CG15109-14-3-3ζ-Idh^34^,^35^,^36^,^37^.) and the ubiquitination pathway (β-tub56D-Rab11-phy1-CG2116-sol-14-3-3ζ-Idh,). In terms of the ubiquitination pathway, many studies have already shown that it is closely related to PD^38^,^39^. However, the relationship between MAPK pathway and Parkinson’s disease is not well understood. So, we next focused on the MAPK pathway to provide an insightful study of the functional relevance of PD.

### LK6 promotes α-synuclein phosphorylation

**Figure 3:**
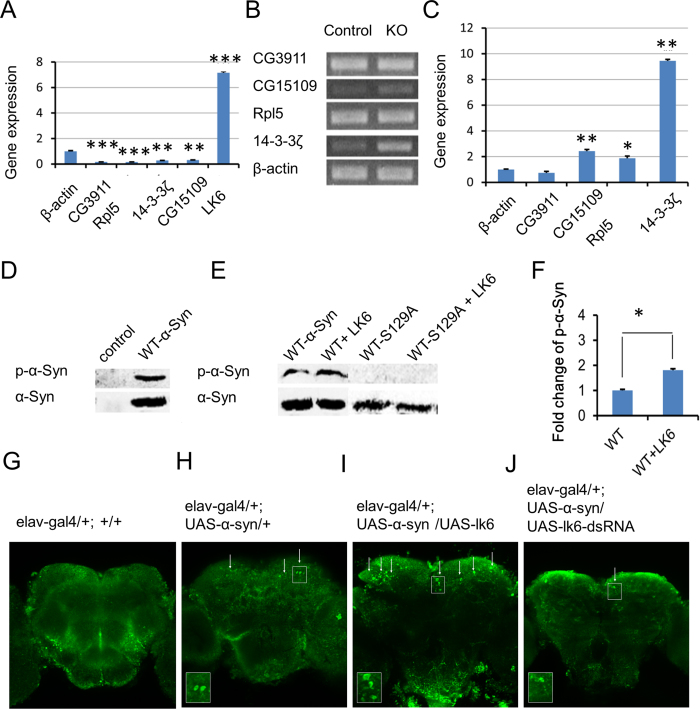
LK6 phosphorylates α-synuclein. (**A**) Quantitative analysis of the expression changes of CG3911, Rpl5, 14-3-3ζ, LK6, CG15109 in the PIN were detected with real-time PCR. (**B**) RT-PCR analysis showing expression of CG3911, Rpl5, 14-3-3ζ, CG15109 between LK6 (-/-) drosophila and wild type (+/+). (**C**) Quantitative analysis of the expression changes of mRNAs in the LK6 (-/-) drosophila. Results are shown as means ± SE of triplicate in three independent experiments. The statistical significance of differences was calculated by using P value (*<0.01; **<0.001; ***<0.0001). (**D**) The expression of endogenous α-synuclein in HEK293T cells. Western blots of protein lysates from cells were transient transfected with WT-α-synuclein and the parental HEK293T cells. (**E**) LK6 phosphorylates WT-α-synuclein and A30P-α-synuclein at Ser129. Phosphorylation was not detected in mutants S129A and S129A-LK6. (**F**) Quantitative results from (**E**) between α-synuclein and α-synuclein–LK6 show a significant increase (n = 3, *p<0.05). Immuno histochemical staining in drosophila brains with antibody indicate a siginificant increase in expressing α-synuclein-LK6(I) than α-synuclein line (**H**) and blank control (**G**). Silence of LK6 by RNAi α-synuclein phosphorylation was significantly decreased (Fig. 3J).

In order to confirm the reliability of the MAPK network (Fig. 2C), we analyzed the expression of CG3911, Rpl5, 14-3-3ζ, LK6, CG15109 by quantitative real-time PCR. Results indicate that LK6 is significantly up-regulated by 7.16-fold in A30P PD mutant (Fig. 3A), suggesting an important role in this network (Fig. 2C). LK6 binds to ERK and is activated by the ERK signaling and it is responsible for phosphorylation of eukaryotic initiation factor 4E (eIF4E) in Drosophila^36^. Arquier *et al.*^40^ also demonstrate that Lk6 exerts a tight control on eIF4E phosphorylation and is necessary for normal growth and development. Consistent with these reports, our experiment indicated a high expression of LK6, suggesting a rescue response in the pathogenesis of PD. On the other hand, CG3911, involved in vesicle-mediated transport, is significantly down-regulated (84%). 14-3-3ζ stimulates the activation of tyrosine hydroxylase (TH). Wang *et al.*^41^ found that 14-3-3zeta knockdown significantly reduces TH activity and dopamine synthesis, with implications for Parkinson disease. Consistent with this result, our research also validated the down-regulation of 14-3-3zeta in α-synuclein mutant, confirming the reliability of our prediction in the PIN. Further, CG15109 is notably decreased (Fig. 3A).

In view of LK6 being highly expressed in the PD mutant, we further explored whether there are changes in LK6-related gene expression when it is knocked out. The results indicate that Rpl5, 14-3-3ζ and CG15109 are upregulated with the exception of CG3911 (Fig. 3B,C), corresponding with the results from the expression of LK6. Together, both LK6 silence and expression confirmed a critical role of LK6 in the network.

Given that LK6 is a kinase, whereas α-synuclein tends to be phosphorylated, Chen *et al.* reported that α-synuclein phosphorylation status is crucial in mediating its neurotoxicity and inclusion formation, which are the characteristic pathologic lesions of Parkinson disease^42^. Since Ser129 in α-synuclein is selectively and extensively phosphorylated in synucleinopathy lesions which are the hallmark lesion in Parkinson’s disease^43^; we therefore explored whether LK6 regulates α-synuclein Ser129 phosphorylation. Due to observations that expression of endogenous α-synuclein in HEK293T cells was too low to be detected by western blot^44^(Fig. 3D), we respectively constructed α-synuclein and LK6 expression vector and co-transfected both of them into HEK293T cells to identify α-synuclein phosphorylation by LK6. Results indicated that the overexpression of LK6 leads to a significant increase of α-synuclein Ser129 phosphorylation (Fig. 3E). Statistical analysis shows a significant difference (p < 0.05) (Fig. 3F). Phosphorylation status is no longer detected in both mutants S129A and S129A-LK6 vice versa (Fig. 3E), indicating LK6 directly phosphorylates α-synuclein. All these results are convincing that LK6 plays a role in promoting α-synuclein Ser129 phosphorylation. *In vivo* tests in drosophila were further confirmed that LK6 significantly enhanced α-synuclein phosphorylation in α-synuclein-LK6 (Fig. 3I) compared with its just α-synuclein-expressing control (Fig. 3H) and blank control (Fig. 3G). Alph-synuclein phosphorylation was obviously reduced in silencing LK6 line by RNAi (Fig. 3J).

### Lk6 promotes degeneration of dopaminergic neuronsand leads to earlier deathin Drosophila

**Figure 4:**
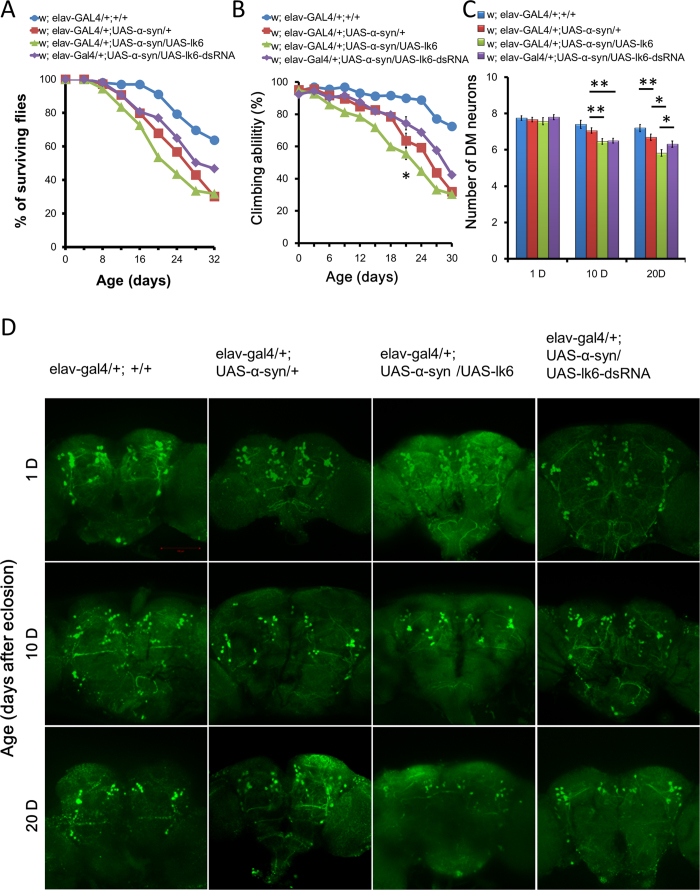
α-synuclein phosphorylation by LK6 shortened drosophila lifespan and caused loss of dopaminergic neurons. (**A**) Quantitative analysis of survival rate in transgenic flies. Data were analyzed by Log-rank (Mantel-Cox) test with *P<0.05, compared to PD flies. Genotypes: control flies are w; elav-gal4/+;+/+. α-synuclein flies are w; elav-gal4/+; UAS-synuclein/+. lk6 overexpression α-synuclein flies are elav-gal4/+; UAS-synuclein/UAS-lk6. lk6 silencing α-synuclein flies are elav-gal4/+; UAS-synuclein/UAS-lk6-dsRNA. (**B**) Overexpression Lk6 accelerates loss of climbing ability in α-synuclein transgenic flies throughout the nervous system (elav-GAL4 driver). Quantitative analysis of climbing ability in transgenic flies. Values represent mean ± SEM. (***P<0.001, compared to wild type flies, ^#^P<0.05, ^##^P<0.01, compared to PD flies, multivariant ANOVA with Bonferroni’s Multiple Comparison Test). Genotypes: control flies are w; elav-gal4/+;+/+. α-synuclein flies are w; elav-gal4/+; UAS-synuclein/+. lk6 overexpression α-synuclein flies are elav-gal4/+; UAS-synuclein/UAS-lk6. lk6 silencing α-synuclein flies are elav-gal4/+; UAS-synuclein/UAS-lk6-dsRNA. (**C**) Confocal images of dopaminergic neurons regions immune stained with anti-TH antibody on 1-d, 10-d, and 20-d fly brains, respectively, with box for DM clusters. A normal number of dorsomedial neurons was identified in 1-d flies in a pan-neural pattern, whereas loss of dopaminergic neurons was observed in 10-d and 20-d α-synuclein transgenic flies with lk6 overexpression. Silence of lk6 could rescue the defect of dopaminergic neurons derived from α-synuclein. Quantitative analysis of TH immunoreactive dorsomedial cluster dopamine neurons in transgenic flies. Values represent mean ± SEM. (*P<0.05, multivariant ANOVA with Bonferroni’s Multiple Comparison Test) .(**D**) Quantitative analysis of dopaminergic neuron numbers over time in the DM clusters ofdifferent genotypes of α-synuclein flies.

To further explore the influence of Lk6 overexpression in organisms, we used the GAL4-UAS system to overexpress Lk6 in α-synuclein transgenic flies. The results indicated that almost half of the α-synuclein transgenic flies over-expression of lk6 died at the age of 20 (w; elav-gal4/+; UAS-synuclein/UAS-Lk6), while 80% of the control flies (w; elav-gal4/+;+/+) and 70% of the PD flies (w; elav-gal4/+; UAS-synuclein/+) are alive (Fig. 4A). Although the survivorship curve continued to decline 24d after eclosion, over-expression of lk6 in adult nervous system significantly shortened the life span of the α-synuclein transgenic flies. In contrast, PD flies silencing lk6 (w; elav-gal4/+; UAS-synuclein/UAS-Lk6-dsRNA) survived more at the same age of the ones over-expressing lk6, showing a rescue relationship (Fig. 4A). We also tested the climbing ability in different genotypes of drosophila, the similar results in life span were also observed. The climbing ability in lk6 overexpressing flies (w; elav-gal4/+; UAS-synuclein/UAS-Lk6) was significantly decreased about 50% compared with the control (w, elav-Gal4/+, +/+), while the reduced climbing ability could be rescued by silencing of lk6 (w; elav-gal4/+;UAS-synuclein/UAS-Lk6-dsRNA) (Fig. 4B).

We further found normal numbers of dorsomedial dopaminergic neurons in 1-day-old transgenic flies in each genotype, but neurons loss in 10-d flies and 20-d transgenic flies with the co overexpression of Lk6 and wild-type a-synuclein (Fig. 4C). The silence of Lk6 could rescue the loss of dopaminergic neurons (Fig. 4C). Further statistical analysis showed significant difference in the number of DM neurons was detected in the 20-d-old flies of these strains (Fig. 4D). Thess results conclusively confirmed that LK6 indeed causes neurodegeneration *in vivo* and thus provide a new understanding of PD.

### Mnk2a phosphorylates α-synuclein and leads α-synuclein inclusion formation

**Figure 5:**
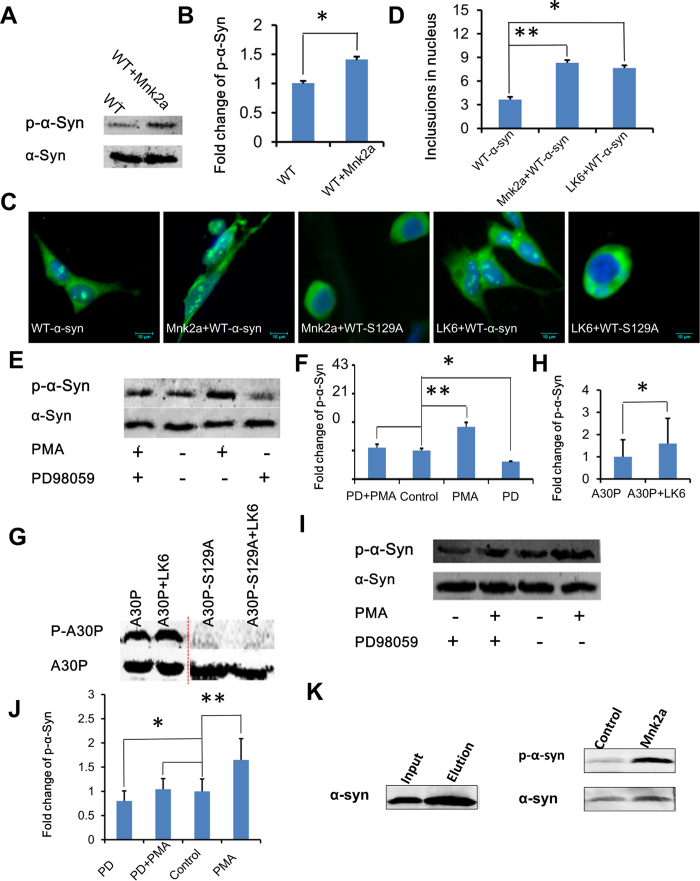
LK6/Mnk2a mediate α-synuclein phosphorylation signal mechanism. (**A**) Mnk2a phosphorylates WT-α-synucleinat Ser129. Quantitative results show a significant increase of α-synuclein phosphorylation level by Mnk2a (**B**). (**C**) Overexpression of Mnk2a and WT-α-synuclein enhanced α-synuclein inclusion formation in nucleus. Cells were transfected with WT-α-synuclein and co-transfected with WT-α-synuclein and Mnk2a or WT-α-synuclein-S129A and Mnk2a. (**D**) Statistical analysis of α-synuclein inclusion formation in nucleus. (**E**) LK6 phosphorylates WT-α-synuclein Ser129 by ERK signaling. Western blots of protein lysates from cells were co-transfected with WT-α-synuclein and LK6. (**F**) Quantitative results of the comparison from (I) showing a 1.83-fold greater capacity for PMA (n = 3, **p<0.01) and a 0.61-fold lower capacity for PD98059 (n = 3, *p<0.05). (**G**) LK6 α-synuclein phosphorylation detection indicates enhanced status in mutant A30P-LK6, whereas phosphorylation disappear in mutants A30P-S129A and A30P-S129A-LK6. Quantitative results confirmed the significant difference between A30P and A30P-LK6 (**H**). (I) Mnk2a phosphorylates WT-α-synuclein Ser129 by ERK signaling. (**J**) Quantitative results of the comparison from (I) showing a 1.65-fold greater capacity for PMA (n = 3, **p<0.01) and a 0.80-fold lower capacity for PD98059 (n = 3, *p<0.05). (**K**) Purified α-synuclein was incubated with cell lysate including overexpressed Mnk2a at concentration of 1 ng/μl with 200 μM ATP for 1 hour. The protein was analyzed by western blotting probed with anti-phospho-α-synuclein and anti α-synuclein antibodies. Cell lysate from 293T overexpressed Mnk2a showed more α-synuclein phosphorylation than control group.

Given that LK6 is genetically similar to human Mnk2a^36^,^40^,^45^,^46^, we further wanted to know whether Mnk2a also phosphorylates α-synuclein. The results indicated that the overexpression of Mnk2a enhanced α-synuclein Ser129 phosphorylation (Fig. 5A). Statistical analysis shows that the difference is significant (p < 0.05) (Fig. 5B).

To validate the physiological importance of α-synuclein phosphorylation by LK6/Mnk2a and its link to Parkinson’s disease, we further argued whether LK6/Mnk2a could be involved in the insoluble α-synuclein aggregate formation in human cell. After co-transfecting with α-synuclein and LK6/Mnk2a into SH-SY5Y cells for 48 h, we observed the formation of α-synuclein granular aggregates in the nucleus (Fig. 5C, Mnk2a-WT-α-Syn, Lk6-WT-α-Syn). Whereas there are less α-synuclein granular aggregatesin the control (Fig. 5C, WT-α-Syn), and there are no aggregates in nucleus in α-synuclein mutant (Fig. 5C Mnk2a-WT-α-Syn-S129A, Lk6-WT-α-Syn-S129A), indicating that α-synuclein phosphorylation at Ser129 by LK6/Mnk2a enhances α-synuclein inclusion formation in SH-5Y5Y cells. Statistical analysis shows a significant difference in numbers of inclusions between Mnk2a, LK6 and the control groups (p < 0.01) (Fig. 5D). These results consist with a previous report that phosphorylation of alpha-synuclein at S129 is critical for the formation of inclusions in PD^21^.

### LK6/Mnk2a α-synuclein phosphorylation through ERK signal pathway

To gain further insights into the possible mechanism regarding α-synuclein Ser129 phosphorylation regulated by LK6, the related signaling pathway was investigated. Previous studies have shown that LK6 can bind stably to ERK^36^. Thus, we made a further investigation by PD 98059, an inhibitor of ERK activation, and PMA, an activator of ERK, to judge whether LK6 phosphorylates α-synuclein Ser129 by ERK signaling. Results indicated that PMA significantly increased Ser129 phosphorylation of α-synuclein (p < 0.01) whereas PD98059 significantly decreased Ser129 phosphorylation of α-synuclein (p < 0.05) (Fig. 5E,F).

By mutation of Ser 129 into Ala 129 (S129A), we found that the phosphorylation of α-synuclein disappeared even in the presence of LK6 (Fig. 3E). Similarly, we examined the α-synuclein Ser129 phosphorylation in A30P regulated by LK6. Results indicated that mutant α-synuclein in A30P was indeed phosphorylated by LK6 (Fig. 5G). Statistical analysis shows a significant difference (p < 0.05) (Fig. 5H). When mutation occured in α-synuclein of A30P (S129A), the phosphorylation of α-synuclein Ser129 was abolished (Fig. 5G).

We also used PMA and PD98059 to explore whether Mnk2a is involved in ERK signaling. Results showed that PMA activated and PD98059 inhibited α-synuclein phosphorylation (Fig. 5I). Statistical analysis showed a significant difference (p < 0.05) (Fig. 5J). As a control comparied with Fig. 5E,I, α-synuclein phosphorylation without LK6/Mnk2 transfection was investigated by treated with PMA and PD98059, showing the results of consistent (S-Fig. 3). These results powerfully confirmed that Mnk2a phosphorylates α-synuclein Ser129 by ERK signal pathway, providing a new insight into the mechanism of PD. Finally, we further identified a direct action of Mnk2a in phosphorylation of α-synuclein by the method *in vitro* kinase assay^47^,^48^. The result showed an increase of α-synuclein phosphorylation by Mnk2 (Fig. 5K, S-Fig. 4).

## Materials and Methods

### Flies and α-synuclein mutants

Tyrosine Hydroxylase (TH)-GAL4 driver, UAS-A30P α-synuclein, UAS-A53T α-synuclein were donated by Dr. AikeGuo, Institute of Neuroscience, Shanghai Institutes for Biological Science, Chinese Academy of Sciences. Virgin females from UAS-A30P α-synuclein and UAS-A53T α-synuclein were crossed to males from TH (tyrosine hydroxylase)-GAL4 driver. This driver line contained the promoter for tyrosine hydroxylase gene drives A30P α-synuclein or A53T α-synuclein expression specifically in dopaminergic neurons. The F1 generation was collected and subjected to further experiments. Drosophila raised at 25 °C were cultured on standard cornmeal medium, and harvested at the same time.

### RNA Isolation, PCR and Q-RTPCR Analysis

Total RNA was isolated from Drosophila heads by using TRIzol (Invitrogen) as described in Ref. 49. Reverse transcription was performed by using ReverTraAce-α (First strand cDNA synthesis kit, TOYOBO). Target gene quantities were normalized to β-actin RNAs. Primers to amplify β-actin, α-synuclein, Tyrosine Hydroxylase (TH), β-tub56D Idh, LK6, 14-3-3ζ, CG3911, CG15109, Rpl5 were described below:

β-actin:

5′ GTCCCAGTTGGTCACGAT 3′

5′ AGTTGCTGCTCTGGTTGT 3′

α-synuclein:

5′ ATGTAGGCTCCAAAACCAAGG 3′

5′ TCCACAGGCATATCTTCCAGAA 3′

TH:

5′ CGCTTTGGTGGTCCGCCTCA 3′

5′ GCAGATTGCCACGGGTCATG 3′

Idh:

5′ CGATGGCAAGACCGTGGAGGC 3′

5′ CTCAAGGGTGTCGGCGAACTG 3′

β-tub56D:

5′ ACCTTCATCGGCAACTTCACT 3′

5′ CTCCTCGAACTCGGCGTCCTC 3′

LK6

5′ CCAGGCGGAGCTTAACAGG 3′

5′ CCTTCACGGCATACTCCAGAT 3′

14-3-3ζ

5′ CAGGTCATCGTGGCGTGTC 3′

5′ CTCGGGATTGCTGGCTTTT 3′

CG3911

5′ CCGATGAGTTCTCCCTGGTATT 3′

5′ CATTGTCGCCCTTCAGTTGAT 3′

CG15109

5′ TGAAGCCGCCACATCTC 3′

5′ CATCAGGCTACCCAAACG 3′

Rpl5

5′ CATGAGCTTCCCAAATACG 3′

5′ ACACGGGCACCAGTTGTAG 3′

### Proteomic Analysis

Two-dimensional gel electrophoresis was done largely according to reference^50^ and the manufacturer’s protocols. For each of three independent replicate experiments of three sets (control and samples), 30 heads from 19-day-old adult Drosophila (half male and half female) of each genotype (control:w1118; samples:UAS-A30P α-synuclein/TH-GAL4 and UAS-A53T α-synuclein/TH-GAL4) were homogenized in 100 μl of lysis buffer (7 M Urea/2M Thiourea/4% CHAPS/2% IPG Buffer/1 mM PMSF/1 mM EDTA/1 mMAprotitin). ImageMaster 2D Platinum 6.0 software was used for attaining a higher level of quantitative and qualitative analysis between two sets of gels. Entries with abundance changes of more than 2-fold increase or decrease were reported and presented for MALDI-TOF-TOF mass spectrometry. Ions specific for each sample were used to interrogate Drosophila sequences deposited in the NCBI data bases using the MASCOT (www.matrixscience.com) search algorithms.

### Construction of protein-protein interactions network

Protein-protein interaction data of Drosophila from DIP^51^,^52^, which includes 7038 proteins and 20720 interactions^53^, were used to construct the Protein-Protein Interactions Network (PIN) of Drosophila in this paper. A human PIN, including 6340 proteins and 23591 interactions between them, came from HPRD^54^. PD related PINs of Drosophila and human were computed and constructed with these two PINs respectively. Graphs were applied to describe the PINs. Each node in graphs represented a protein, and each edge between two nodes denoted the interaction between corresponding proteins. For details, it can be seen at our Website (http://hpca.shu.edu.cn/inm/)

### ERK-specific signal pathway inhibition and activation

HEK293T cells were co-transfected with the plasmids LK6 & WT-α-Syn, LK6 & A30P and Mnk2a & WT-α-Syn. After 24 h, cells were serum-starved for 16 h and subsequently harvested directly with the signaling inhibitor PD98059 and the activator PMA for 1 h and 45 min respectively at final concentrations of 50 and 10 μM respectively. To contrast, we also designed a group that, after 24 h transfection, cells were serum-starved for 16 h and subsequently treated with PD98059 after preincubation of the cells for PMA^55^.

### Western blotting

Cells were lysed in RIPA-50 buffer (50 mMTris–HCl, 150 mMNaCl, 1% NP-40, 1 mMEDTA, 0.1% SDS and 0.01% NaN3, 1 mMPMSF, pH 7.4). Equal amounts of proteins were separated by SDS-PAGE on 10% polyacrylamide gels and electroblotted onto nitrocellulose membrane. Membranes were blocked overnight at 4 °C in Odyssey blocking buffer and simultaneously incubated with a rabbit anti-Syn antibody EP1646Y (1:1000, Epitomics) and a rabbit anti- pS129-Syn antibody EP1536Y (1:1000, Epitomics) for 1 h at room temperature with gentle shaking. Next, infrared dye 800-conjugated affinity-purified goat anti-rat IgG (Zemed, USA) secondary antibodies were added and allowed to incubate for 1 h at room temperature. Immunoblotting bands were detected and quantified utilizing the LI-COR Odyssey infrared imaging system and software (LI-COR).

### Immunocytochemistry

Cells were seeded over night, at 70% confluence, onto cover slips in 24-well dishes and transfected with the appropriate plasmids the following day for 48 h. After washing with phosphate-buffered saline, the cells were fixed with neutral buffered 4% (w/v) paraformaldehyde for 15 min and permeabilized with 0.1% TritonX-100 for 30 min and then blocked with 5% goat serum. Cells were incubated at 4 °C for 16 h with the appropriate primary antibody. After washing with phosphate-buffered saline, FITC-secondary antibodies were addedand incubated for 2 h at room temperature. After incubating cells with DAPI for 15 min, fixed cells were analyzed by confocal or fluorescence microscopy.

### *In vitro* kinase assay

Purified α-synucleinwas incubated with cell lysate including overexpressed Mnk2a at concentration of 1 ng/μlin kinase buffer (25 mMTris-HCl, pH 7.5, 5 mM β-glycerophosphate, 2 mM dithiothreitol, 0.1 mM Na3VO4, 0.1 mMNaF, 10 mM MgCl2, 200 μM ATP) for 1 hour. The protein from both groups was subjected to SDS-PAGE and western blotting.

## Discussion

This is a comprehensive study from *in vitro* to *in vivo*, from theory to experiment. The major findings are as follows: (1) LK6 plays a pivotal role in α-synuclein phosphorylation and α-synuclein inclusion (Lewy body) formation. (2) Mnk2a, a homologous protein of LK6, also caused α-synuclein inclusion formation in human and mouse neurons, which is directly related to PD, providing a new insight into the mechanism of PD. (3) *In vivo* tests in LK6 drsophila were demonstrated a significant lose of dopaminergic neurons, a decrease in the life span and climbing ability with overexpression of LK6.

For the study of Parkinson’s disease is more than two hundred years. In the meantime, countless patients with PD lose their lives helplessly. One of the main reasons in the clinical treatment is the lack of understanding of the pathogenesis of PD. There are different ways to explore the pathogenesis, but inevitably, it must be clearly recognized the short comings in present approach. In order to avoid the shortage of current method as much as possible, we need to change the traditional ideas to find a way to search pathogenesis of PD. In this study, we constructed a comprehensive protein interaction network based on α-synuclein A30P and A53T mutation in a systematic and integrated view to support of a greater understanding at a systems biology level from drosophila to human. In contrast to previous articles which are limited to a single mutation, we take full advantage of all published protein library data to give an overall understanding of PD. This is probably by far the most comprehensive and integrated thinking to study PD.

The most striking innovation in this report is for the first time found that the LK6 kinase regulated signaling pathway involving α-synuclein phosphorylation, suggesting that LK6 plays a pivotal role in the formation of PD. As a homologous protein of LK6, Mnk2a is found to phosphorylates α-synuclein and causes α-synuclein inclusion Lewy body formationin human and mouse neurons, which is directly related to PD, providing a new insight into the mechanism of PD. *In vivo* tests in LK6 drsophila were demonstrated a significant lose of dopaminergic neurons, a reduce in the life span and climbing ability with overexpression of LK6, which are typical feature of PD^56^. LK6 or Mnk2a mediates this process by ERK signaling, suggesting a potential drug target. It will provide a new basis for the treatment of PD.

The merit of the approach is to provide a large-scale analysis of protein interactions, and characterize the different protein function in a level of network. The real functional molecules are being studied during metabolic processes. Without this way, we will be like searching for a needle in a haystack to determine the connection among the different proteins. However, we acknowledge that any methods have their short comings, as we have seen, the proteomic analysis fails to detect LK6, even its mRNA is increased 7.16-fold in PD mutant. This does not hinder us to use this method. By combining biological experiments, we made up for this shortage, providing a new understanding of PD.

Overall, these studies provided highly integrated information from drosophila to human based on α-synuclein mutation. We revealed and confirmed the protein interaction network by a combination of systems biology methods and biological experiments. Our study firstly showed that LK6 phosphorylates α-synuclein Ser129 by ERK signaling and more importantly showed that Mnk2a leads to α-synuclein inclusion formation by phosphorylation of α-synuclein at Ser129 in human. This study provides a novel insight into the mechanism for PD pathogenesis, and also suggestes a possible target for PD diagnosis in the future.

## Additional Information

**How to cite this article**: Zhang, S. *et al.* LK6/ Mnk2a is a new kinase of alpha synuclein phosphorylation mediating neurodegeneration. *Sci. Rep.* **5**, 12564; doi: 10.1038/srep12564 (2015).

## Supplementary Material

Supplementary Information
