# Supplementary material for: LK6/Mnk2a is a new kinase of alpha synuclein phosphorylation mediating neurodegeneration
Source: Sci Rep. 2015 Jul 29;5:12564. doi: 10.1038/srep12564 (PMC4518213; doi:10.1038/srep12564)
Supplement: Supplementary Information [file srep12564-s1.pdf]

Supporting figures and tables

LK6/ Mnk2a is a new kinase of alpha synuclein phosphorylation mediating neurodegeneration

Shiqing Zhang[1,2,*], Jiang Xie[3,*], Ying Xia[1,*], Shu Yu[1,*], Zhili Gu[1,*], Ruili Feng[1], Guanghong Luo[1], Dong Wang[1], Kai Wang[1,2], Meng Jiang[1], Xiao Cheng[3], Hai Huang[1], Wu Zhang[3], Tieqiao Wen[1,2 †]

**: These authors contributed equally to this work*
†: To whom correspondence may be addressed. E-mail:tqwen@staff.shu.edu.cn; Tel:86-021-66133819; Fax: 86-021-66136129

Sup-figure legends

S-Fig. 1 Silver-stained 2DE gels of Drosophila brain proteins. Representative 2D gels of proteins from brains of w1118 Drosophila 19 days after eclosion (A, C), of α-synuclein A53T Drosophila day 19 after eclosion (B) and of α-synuclein A30P Drosophila 19 days after eclosion (D) are shown.

S-Fig. 2 Differential analysis of β-tub56D, Idh and CG2233 in silver-stained 2DE gels. The expression of β-tub56D is up-regulated in α-synuclein A53T/A30P expressing Drosophila (A-F). The expression of Idh is down-regulated in α-synuclein A53T Drosophila (G-I). The expression of Idh is up-regulated in α-synuclein A30P Drosophila (J-L).The expression of 3 isoforms of unknown protein CG2233 is down-regulated in α-synuclein A53T Drosophila (M-O). Yellow vertical lines stand for the concentration of the proteins (measured as density at vol. %) Red lines mean the highest and lowest concentration of the proteins, and blue lines represent the average value. Spots were highlighted in green.

S-Fig. 3 α-synuclein phosphorylation activity. α-synuclein phosphorylation was tested in treatment with PMA and PD98059 without LK6/Mnk2 transfection. “+” or “-”represent addition or no addition of PMA or PD98059 respectively.

S-Fig. 4 Purification and in vitro kinase activity assay of α-synuclein and MNK2A. (A) Immunoaffinity purification of α-synuclein and MNK2A complex from HEK293T cells co-transfected with PCAGGS-a-synuclein-His and N2-mnk2a-1D4. Solubilized HEK293 cell extract (Input) incubated with the Ni SepharoseTM 6 matrix, and the eluted fractions (Elution) were analyzed by western blotting probed with the α -His (a-synuclein) andα -1D4 (MNK2A) antibody. Co-expression and co-immunoprecipitation studies indicated that a-synuclein and MNK2A can be purified. (B) Purified α-synuclein/MNK2A were incubated in the present of 50mM Hepes, PH7.5, 150mM NaCl, 12.5mM MgCl2, 1mM DTT at concentration of 1ng/ul protein with (+) or without (-) 200 uM ATP for 1 hour. The protein was analyzed by western blotting probed with anti-phospho-α-synuclein and anti α-synuclein antibodies. The purifiedα-synuclein/MNK2A complex showed more α-synuclein phosphorylation with ATP than control group (without ATP).

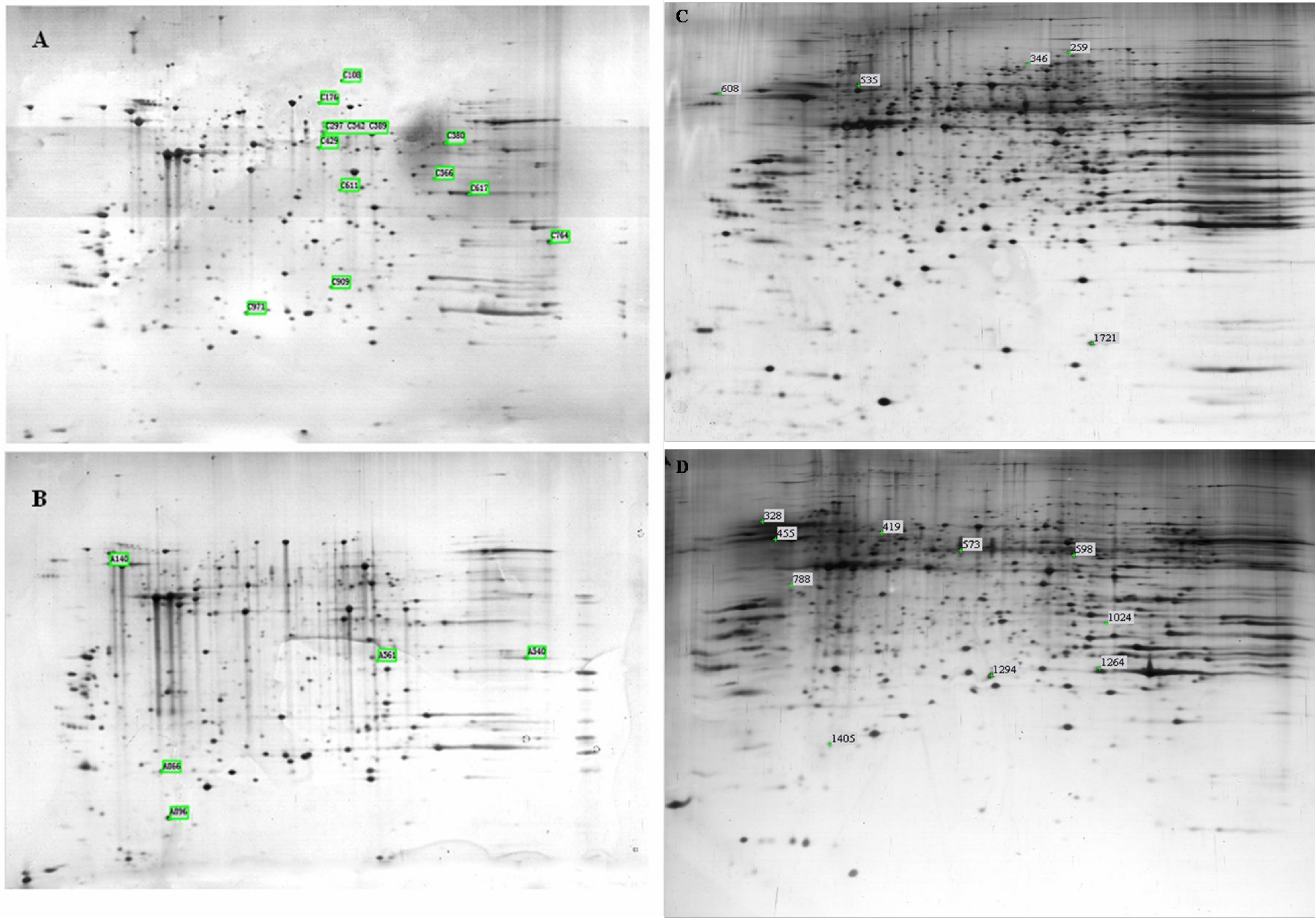
A
C108
C176
C297 C342 C389
C429
C380
C366
C611
C617
C764
C909
C971
B
A140
A561
A540
A866
A896
C
259
346
535
608
1721
D
328
455
419
573
598
788
1024
1294
1264
1405

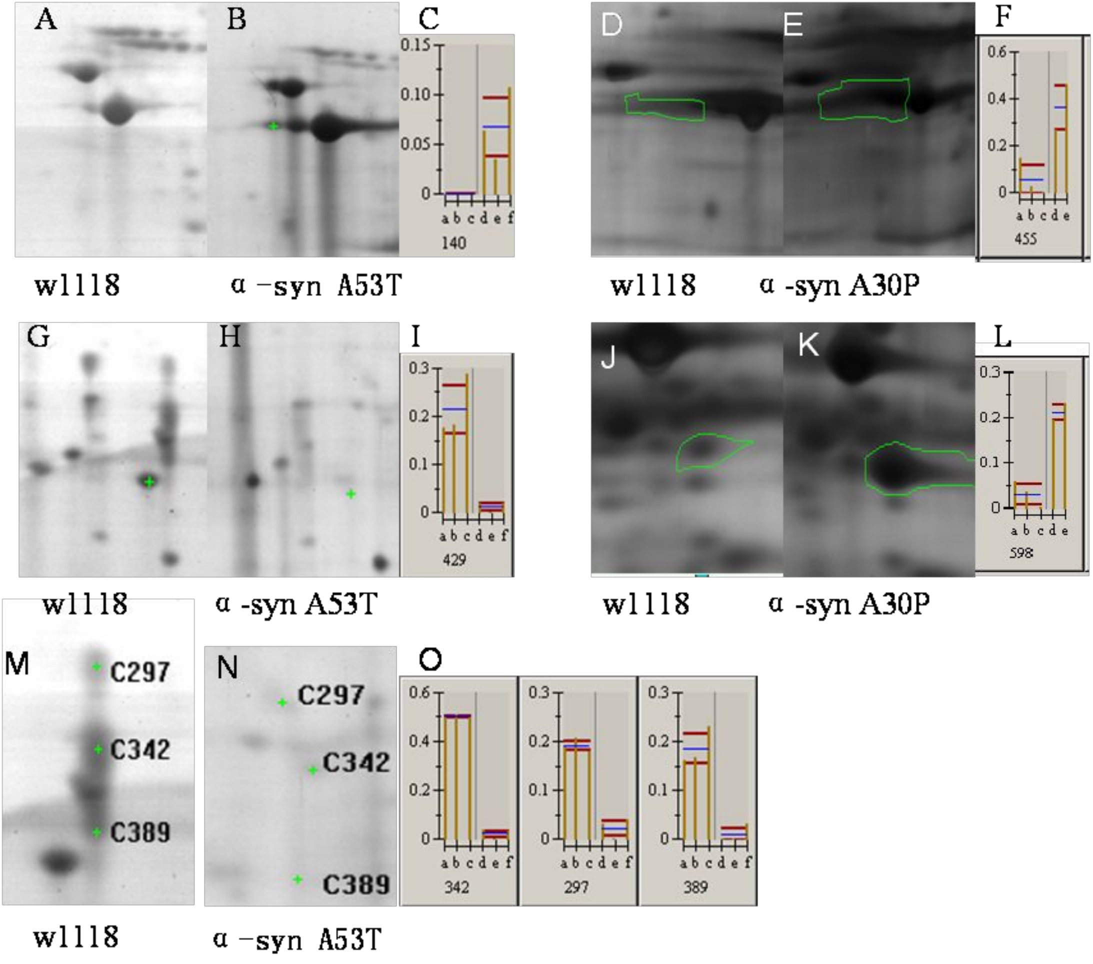

A
B
C
140
w1118
α -syn A53T
D
E
F
455
w1118
α -syn A30P
G
H
I
429
w1118
α -syn A53T
J
K
L
598
w1118
α -syn A30P
M
C297
C342
C389
w1118
N
C297
C342
C389
α -syn A53T
O
342
297
389

S - Figure 3

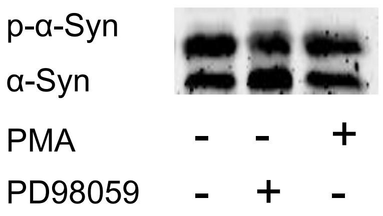

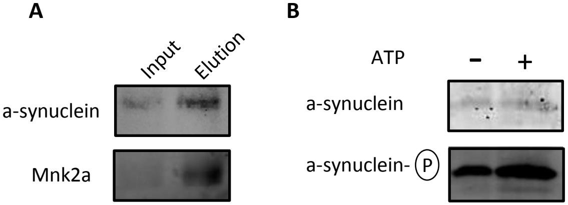
A
Input
Elution
a-synuclein
Mnk2a
B
ATP
-
+
a-synuclein
a-synuclein- P

S-Table 1

Altered protein expression in α-syn A53T mutant brain		
Protein	ID	Change
Mitochondrial Proteins		
Aldehyde dehydrogenase (CG3752)	C176	-10000
Isocitrate dehydrogenase (CG7176)	C429	-8.09
CG7998	C764	-3.52
CG3609	C566	-4.57
Ferritin 1 heavy chain homologue (CG2216)	A896	+10000
Cytoplasm		
Glycerol 3 phosphate dehydrogenase (CG9042)	A561	-2.20
Glyceraldehyde phosphate dehydrogenase (CG8893)	A540	+10000
Aldolase (CG6058)	C611	-2.98
Peroxiredoxin 5037 (CG5826)	C971	-2.93
Lysosome		
Peroxiredoxin 2540 (CG11765)	C909	-3.99
Proteasome 35kD subunit (CG4904)	C617	-10000
Cytoskeleton proteins		
β-Tubulin at 56D (CG9277)	A140	+10000
Unknow proteins		
CG2233	C297	-4.63
	C342	-13.43
	C389	-6.31
Miscellaneous		
Yolk protein 3 (CG11129)	C380	-3.12
Retinin (CG13057)	A866	+3.40
LD13607p (CG12236)	C108	-3.19

+, increased expression; -, decreased expression. +10000, the protein only exists in α-syn A53T brains; -10000, the protein only exists in w1118 brains.

S-Table 2

Altered protein expression in α-syn A30P mutant brain		
Protein	ID	Change
Mitochondrial Proteins		
Isocitrate dehydrogenase(CG7176)	A598	+3.53
Succinyl coenzyme A synthetase flavoprotein subunit(CG17246)	C346	-10000
Hsp60C(CG7235)	A1264	+2.69
Triose phosphate isomerase(CG2171)	A1294	+7.35
ATP synthase-β(CG11154)	A455	+2.22
Extracellular		
CG32068	A1405	+2.70
ER Proteins		
Calreticulin(CG9429)	C608	-10000
Nuclear proteins		
CG1119	A419	+2.61
Cytoskeletal Proteins		
β-Tubulin at 56D(CG9277)	A455	+2.22
twinstar(CG4254)	C1721	-10000
dilute class unconventional myosin(CG2146)	A328	+10000
Membrane proteins		
Tie-like receptor tyrosine kinase(CG7525)	A328	+10000
Unknown		
CG8108	A1024	+2.13
Miscellaneous		
Yolk protein 1(CG2985)	A573	+2.17
Crystallin(CG16963)	C535	-10000
Black cells(CG5779)	C259	-10000
Cuticular protein 67B (CG3672)	A788	+10000

+, increased expression; -, decreased expression. +10000, the protein only exists in α-syn A30P brains; -10000, the protein only exists in w1118 brains.

S-table 3

Corresponding relations of the proteins in networks of of α-synuclein A53T in Drosophila (Fig. 1A) and Human background (Fig. 1C)

Drosophila		Human		Drosophila		Human	
Locus Tag	Genebank ID	Uniprot ID	Genebank ID	Locus Tag	Genebank ID	Uniprot ID	Genebank ID
CG9042	Gpdh	P21695	GPD1	CG4071	vsp20	P53350	PLK1
CG8893	Gapdh2	P04406	GAPDH	CG3081	CG3081	Q96CW5	TUBGCP3
CG7176	Idh	O75874	IDH1	CG5216	Sir2	O14939	PLD2
CG6058	Ald	P04075	ALDOA	CG10327	TBPH	O14576	DYNC1I1
CG2216	Fer1HCH	P02794	FTH1	CG14534	TwdlE	Q99062	CSF3R
CG11765	Prx2540-2	P30041	PRDX6	CG15141	CG15141	Q03164	MLL
CG9277	β Tub56D	P23258	TUBG1	CG4832	cnn	P49023	PXN
CG2233	CG2233	O95487	SEC24B	CG15737	CG15737	Q96L34	MARK4
CG14025	Bsg25D	P54257	HAP1	CG13287	CG13287	O00459	PIK3R2
CG11581	CG11581	Q9BX70	BTBD2	CG10943	CG10943	O14490	DLGAP1
CG10685	I(2)37Cg	O00299	CLIC1	CG4853	CG4853	P38398	BRCA1
CG7357	CG7357	P27986	PIK3R1	CG10034	tj	O14933	UBE2L6
CG11656	CG11656	O75822	EIF3J	CG7399	Hn	P02792	FTL
CG13214	Cpr47Ef	O43504	HBXIP	CG30122	CG30122	P21980	TGM2
CG32096	rols	Q92834	RPGR	CG9335	CG9335	O95613	PCNT
CG13030	CG13030	P10275	AR	CG17342	Lk6	Q07002	PCTK3
CG9876	CG9876	O15287	FANCG	CG14228	Mer	P21246	PTN
CG18362	Mio	Q14194	CRMP1	CG11380	CG11380	P41743	PRKCI
CG17233	CG17233	O00231	PSMD11	CG9331	CG9331	P05783	KRT18
CG14207	CG14207	O43353	RIPK2	CG12470	CG12470	P00533	EGFR
CG15071	CG15071	Q8NBJ4	GOLM1	CG13510	CG13510	Q99972	MYOC
CG16747	Oda	P63167	DYNLL1	CG10695	Pat1	O00145	INPP5D
CG15224	CkIIβ	Q9NV58	RNF19A	CG32016	CG32016	P51116	FXR2
CG14829	CG14829	O15169	AXIN1	CG10108	plyl	O14976	GAK
CG14718	CG14718	P43304	GPD2	CG9610	Poxm	P40925	MDH1
CG8232	CG8232	O60861	GAS7	CG2934	VhaAC39	P36543	ATP6V1E1
CG5099	msi	Q9UNP9	PPIE	CG10364	msb1l	Q13368	MPP3
CG1472	sec24	Q15436	SEC23A	CG33070	CG14425	Q13748	TUBA3C
CG8597	lark	P08727	KRT19	CG17870	14-3-3ζ	Q92793	CREBBP
CG4071	vsp20	P53350	PLK1	CG33103	Ppn	P14209	CD99
				CG11230	CG11230	Q9UKY1	ZHX1

S-table 4

Corresponding relations of the proteins in networks of of α-synuclein A30P in Drosophila (Fig. 1B) and Human background (Fig. 1D)

Drosophila		Human		Drosophila		Human	
Locus Tag	Genebank ID	Uniprot ID	Genebank ID	Locus Tag	Genebank ID	Uniprot ID	Genebank ID
CG14206	RpS10b	P02787	TF	CG14868	CG14868	P13861	PRKAR2A
CG1378	tll	O00585	CCL21	CG9429	Crc	P27797	CALR
CG33070	CG33070	O95477	ABCA1	CG7787	CG7787	P04637	TP53
CG18371	CG18371	P01106	MYC	CG8173	CG8173	Q96L34	MARK4
CG32100	CG32100	Q01844	EWSR1	CG2146	didum	Q9Y4I1	MYO5A
CG8524	NK7.1	P43699	NKX2-1	CG6972	CG6972	O14920	IKBKB
CG13287	CG13287	P06396	GSN	CG3911	CG3911	Q9UKY1	ZHX1
CG18290	Act87E	P54253	ATXN1	CG13379	Sgf11	P04629	NTRK1
CG9665	Cpr73D	P08727	KRT19	CG10531	Cht9	Q9GZR7	DDX24
CG4835	CG4835	P61289	PSME3	CG5093	CG5093	O15379	HDAC3
CG11719	Mst98Ca	P08833	IGFBP1	CG6996	CG6996	Q16820	MEP1B
CG12081	CG12081	P51610	HCFC1	CG9331	CG9331	P38398	BRCA1
CG7518	CG7518	O15162	PLSCR1	CG5094	Sgt	Q9BV36	MLPH
CG7835	Mnb	P16870	CPE	CG8229	CG8229	P00747	PLG
CG1544	CG1544	Q02218	OGDH	CG5164	GstE1	P36542	ATP5C1
CG3644	bic	O96019	ACTL6A	CG8640	Cpr65Ea	O15533	TAPBP
CG4491	noc	P06241	FYN	CG14904	Scp2	O00222	GRM8
CG4865	dgt4	P51671	CCL11	CG5725	fbl	O75083	WDR1
CG15141	CG15141	O15117	FYB	CG3201	CG3201	O15530	PDPK1
CG32852	CG32852	O94973	AP2A2	CG6203	Fmr1	O00273	DFFA
CG6958	Nup133	O15111	CHUK	CG7176	Idh	O75874	IDH1
CG7524	Src64B	Q96CW5	TUBGCP3	CG6667	dl	O14495	PPAP2B
CG11154	ATPsyn-β	P06576	ATP5B	CG4254	RpLP2	P23528	CFL1
CG15436	CG15436	Q9H8E8	CSRP2BP	CG8292	CG8292	O14776	TCERG1
CG10954	Arc-p34	P04114	APOB	CG10139	CG10139	O95613	PCNT
CG9415	Xbp1	P38936	CDKN1A	CG13780	Pvf2	P08047	SP1
CG9040	CG9040	O75382	TRIM3	CG12496	CG12496	Q99996	AKAP9
CG3052	HLH4C	O14604	TMSB4Y	CG1478	Cp36	O14512	SOCS7
CG30084	Zasp	P07948	LYN	CG1464	ey	Q92598	HSPH1
CG7763	CG7763	O00459	PIK3R2	CG6617	CG6617	P51159	RAB27A
CG5798	CG5798	P02647	APOA1	CG9121	CG9121	P53350	PLK1
CG32951	CG32951	P14672	SLC2A4	CG17534	GstE9	O15294	OGT
CG15224	CkIIβ	Q16512	PKN1	CG9335	CG9335	P27986	PIK3R1
CG7845	CG7845	P00451	F8	CG4810	CG4810	O60674	JAK2
CG13200	CG13200	O14939	PLD2	CG4832	cnn	Q13748	TUBA3C
CG7761	pcs	Q70J99	UNC13D	CG2116	CG2116	O43150	ASAP2
CG17107	CG17107	Q15569	TESK1	CG8461	Act88F	O14746	TERT
CG6801	l(3)j2D3	O15226	NKRF	CG1307	CG1307	P05106	ITGB3
CG31363	Jupiter	P53671	LIMK2	CG14204	CG14204	P05067	APP
CG8597	RhoGAP92B	P68371	TUBB2C	CG10327	CG10327	P30443	HLA-A
CG8813	TBPH	O00221	NFKBIE	CG4059	ftz-f1	O75791	GRAP2
CG32171	Lmpt	P01100	FOS	CG7235	Hsp60C	P10809	HSPD1

CG32139	Sox21b	Q05066	SRY	CG6156	CG6156	P63167	DYNLL1
CG17870	CG9455	Q9BQ66	KRTAP4-12	CG1119	Gnf1	P35251	RFC1
CG5771	Rab11	O60269	GPRIN2	CG4755	lark	O00299	CLIC1
CG15737	CG15737	P49023	PXN	CG6569	CG6569	Q92614	MYO18A
CG10377	Hrb27C	P04150	NR3C1	CG14224	Ubqn	Q8N726	CDKN2A
CG12470	CG12470	P42574	CASP3	CG32810	CG32810	P53667	LIMK1
CG4853	CG4853	O15392	BIRC5	CG5203	CHIP	P55210	CASP7
CG10619	tup	O14757	CHEK1	CG3099	CG3099	P01011	SERPINA3
CG10108	Nha2	O43597	SPRY2	CG3610	CG3610	Q9C040	TRIM2
CG11354	CG11354	Q16527	CSRP2	CG7069	CG7069		
CG11768	Mlc-c	Q96S53	TESK2	CG9470	MtnA		
CG4918	CG4918	P29353	SHC1	CG9797	CG9797		
CG4778	obst-B	P00750	PLAT	CG31820	CG31820		
CG13338	Cpr50Ca	O00160	MYO1F	CG9099	CG9099		
CG14850	Doc3	O00311	CDC7	CG14667	CG14667		
CG10699	CG10699	Q04206	RELA	CG3647	stc		
CG4264	Hsc70-4	P10275	AR	CG31319(CG34389)	cv-c		
CG10293	how	P05412	JUN	CG4209	CanB		
CG8277	eIF4E-5	Q92834	RPGR	CG11656	CG11656		
CG8057	alc	O15519	CFLAR	CG10559	CG10559		
CG17377	CG17377	O95838	GLP2R	CG17769	And		
CG11761	trsn	Q99417	MYCBP	CG6945	CG6945		
CG12362	CG12362	Q9Y4X5	ARIH1	CG17342	Lk6		
CG14243	TwdlD	P11021	HSPA5	CG18372	AttB		
CG16972	CG16972	O95782	AP2A1	CG15482	CG15482		
CG5779	Bc	O14727	APAF1	CG7357	CG7357		
CG18396	Mst98Cb	P02786	TFRC	CG3129	Rab-RP4		
CG4735	Shu	O43823	AKAP8	CG4771	CG4771		
CG15634	CG15634	O15287	FANCG	CG32068	CG32068		
CG3114	ewg	O00590	CCBP2	CG9473	MED6		
CG3924	Chi	O75925	PIAS1	CG4325	CG4325		
CG9277	βTub56D	P23258	TUBG1	CG4963	CG4963		
CG3918	CG3918	O00499	BIN1	CG5064	Srp68		
CG11173	usnp	O43293	DAPK3	CG8316	CG8316		
CG5178	Mer	P05023	ATP1A1	CG3295	CG3295		
CG8506	Rbsn	P41219	PRPH	CG11092	CG11092		
CG2985	Yp1	P01350	GAST	CG2021	CG2021		
CG7849	CG7849	Q9NV58	RNF19A	CG1374	tsh		
CG11094	dsx	P01275	GCG	CG31958	CG31958		
CG5494	Cpr92F	O60573	EIF4E2	CG6985	CG6985		
CG9285	Dip-B	P08473	MME	CG12379	CG12379		
CG10034	tj	O60583	CCNT2	CG10685	l(2)37Cg		
CG15067	CG15067	P00533	EGFR	CG17776	CG17776		
CG7525	Tie	Q02763	TEK	CG13997	Fcp26Ac		
CG6315	fl(2)d	P63261	ACTG1	CG15706	CG15706		
CG5009	Lim3	O15151	MDM4	CG4896	CG4896		
CG14546	CG14546	Q9NS73	MBIP	CG14853	CG14853		

CG8614	Neos	P07196	NEFL	CG4753	CG4753
CG14112	CG14112	P04626	ERBB2	CG10340	tsr
CG14228	CG14228	O43524	FOXO3	CG10943	CG10943
CG13383	CG13383	P03372	ESR1	CG14829	CG14829
CG32159	CG32159	P02675	FGB	CG17246	Scs-fp
CG3081	CG3081	P56945	BCAR1	CG14718	CG14718
CG12932	brp	O75444	MAF	CG14820	CG14820
CG15136	CG15136	P51531	SMARCA2	CG30122	CG30122
CG9455	14-3-3ζ	O00145	INPP5D	CG14025	Bsg25D
CG32096	rols	P00450	CP	CG4071	Vps20
CG15781	CG15781	Q9Y234	LIPT1	CG13549	yip3
CG11466	Cyp9f2	P06213	INSR	CG5053	CG5053
CG9986	CG9986	O00194	RAB27B	CG14545	Lim1
CG31052	phyl	P24928	POLR2A	CG14247	CG14247
CG15443	CG15443	Q15047	SETDB1	CG16721	CG16721
CG9975	CG9975	P05783	KRT18	CG13603	CG13603
CG10695	Pat1	P02545	LMNA	CG16985	CG16985
CG33209	CG33209	O75342	ALOX12B	CG11230	SNCF
CG2171	Tpi	P60174	TPI1	CG15649	CG15649
CG8108	CG8108	Q9ULV3	CIZ1	CG5232	Sas

S-Table5

Corresponding relations of the proteins in networks between CG9277 and CG7176 in Drosophila (Fig.2 A) and Human background (Fig.2 B)

Locus Tag	Genebank ID	Uniprot ID	Genebank ID	Locus Tag	Genebank ID	Uniprot ID	Genebank ID
CG9277	βTub56D	P23258	TUBG1	CG11354	Lim1	Q14258	TRIM25
CG7176	Idh	O75874	IDH1	CG32096	rols	Q96CW5	TUBGCP3
CG17870	14-3-3ζ	P63104	YWHAZ	CG7761	pcs	P27986	PIK3R1
CG15649	CG15649	Q9UKY1	ZHX1	CG8277	eIF4E-5	P06730	EIF4E
CG14868	CG14868	O00471	EXOC5	CG40127	CG40127	O15530	PDPK1
CG9335	CG9335	P08727	KRT19	CG4853	CG4853	P49023	PXN
CG9392	CG9392	O14978	ZNF263	CG10943	CG10943	O14776	TCERG1
CG7524	Src64B	P33981	TTK	CG17342	Lk6	Q96L34	MARK4
CG13030	sinah	O14818	PSMA7	CG2116	CG2116	O60260	PARK2
CG10108	phyl	P68036	UBE2L3	CG8907	CG8907	O75190	DNAJB6
CG14667	CG14667	O00231	PSMD11	CG6459	CG6459	O15287	FANCG
CG15141	CG15141	Q05086	UBE3A	CG7461	CG7461	O43924	PDE6D
CG15109	CG15109	P10636	MAPT	CG4832	cnn	P53350	PLK1
CG9184	CG9184	P04406	GAPDH	CG14025	Bsg25D	P35580	MYH10
CG33070	Sex lethal	Q92834	RPGR	CG4767	Tektin-A	P35579	MYH9
CG10686	tral	O60383	GDF9	CG3099	CG3099	P46934	NEDD4
CG16747	Oda	P06748	NPM1	CG9797	CG9797	O95613	PCNT
CG11230	CG11230	P04637	TP53	CG1263	RpL8	Q99996	AKAP9
CG13287	CG13287	O14641	DVL2	CG8470	mRpS30	P20160	AZU1
CG4087	RpLP1	O14965	AURKA			P37840	SNCA
CG15482	CG15482	O00499	BIN1	CG2233	CG2233	O95487	SEC24B
CG4755	RhoGAP92B	P68371	TUBB2C	CG5099	msi		
CG5771	Rab11	P38398	BRCA1	CG10034	tj		
CG12362	CG12362	Q9NV58	RNF19A	CG10364	msb1l		
CG4325	CG4325	Q13748	TUBA3C	CG1472	sec24		
CG3911	CG3911	P60953	CDC42			P53992	SEC24C
CG32276	CG32276	O43150	ASAP2			Q07002	PCTK3
CG1391	sol	O14933	UBE2L6			Q15834	CCDC85B
CG9665	Cpr73D	P05783	KRT18			Q00536	PCTK1
CG11377	CG11377	O75914	PAK3			Q04917	YWHAH
CG17489	RpL5	P05129	PRKCG			Q00535	CDK5
CG10327	TBPH	P19338	NCL			P17612	PRKACA
